# HLA-based banking of induced pluripotent stem cells in Saudi Arabia

**DOI:** 10.1186/s13287-023-03612-0

**Published:** 2023-12-18

**Authors:** Maryam Alowaysi, Robert Lehmann, Mohammad Al-Shehri, Moayad Baadhaim, Hajar Alzahrani, Doaa Aboalola, Asima Zia, Dalal Malibari, Mustafa Daghestani, Khaled Alghamdi, Ali Haneef, Dunia Jawdat, Fahad Hakami, David Gomez-Cabrero, Jesper Tegner, Khaled Alsayegh

**Affiliations:** 1 King Abdullah International Medical Research Center (KAIMRC), King Saud Bin Abdulaziz University for Health Sciences, Ministry of National Guard for Health Affairs, Jeddah, Saudi Arabia; 2 Biological and Environmental Science and Engineering Division, King Abdullah University of Science and Technology (KAUST), Thuwal, Saudi Arabia; 3 Molecular Medicine Section, Department of Pathology and Laboratory Medicine, Ministry of the National Guard - Health Affairs, Jeddah, Saudi Arabia; 4 Forensic Laboratories, Criminal Evidence Department, Jeddah, Saudi Arabia; 5 Computer, Electrical and Mathematical Sciences and Engineering Division, King Abdullah University of Science and Technology (KAUST), Thuwal, Saudi Arabia

**Keywords:** Induced pluripotent stem cells, HLA-based banking, IPS-based therapies, Biobanking, Saudi Arabia

## Abstract

**Background:**

Human iPSCs' derivation and use in clinical studies are transforming medicine. Yet, there is a high cost and long waiting time associated with autologous iPS-based cellular therapy, and the genetic engineering of hypo-immunogenic iPS cell lines is hampered with numerous hurdles. Therefore, it is increasingly interesting to create cell stocks based on HLA haplotype distribution in a given population. This study aimed to assess the potential of HLA-based iPS banking for the Saudi population.

**Methods:**

In this study, we interrogated the HLA database of the Saudi Stem Cell Donor Registry (SSCDR), containing high-resolution HLA genotype data from 64,315 registered Saudi donors at the time of analysis. This database was considered to be a representative sample of the Saudi population. The most frequent HLA haplotypes in the Saudi population were determined, and an in-house developed iterative algorithm was used to identify their HLA matching percentages in the SSCDR database and cumulative coverage. Subsequently, to develop a clinically relevant protocol for iPSCs generation, and to illustrate the applicability of the concept of HLA-based banking for cell therapy purposes, the first HLA-based iPS cell line in Saudi Arabia was generated. Clinically relevant methods were employed to generate the two iPS clones from a homozygous donor for the most prevalent HLA haplotype in the Saudi population. The generated lines were then assessed for pluripotency markers, and their ability to differentiate into all three germ layers, beating cardiomyocytes, and neural progenitors was examined. Additionally, the genetic stability of the HLA-iPS cell lines was verified by comparing the mutational burden in the clones and the original blood sample, using whole-genome sequencing. The standards set by the American College of Medical Genetics and Genomics (ACMG) were used to determine the clinical significance of identified variants.

**Results:**

The analysis revealed that the establishment of only 13 iPSC lines would match 30% of the Saudi population, 39 lines would attain 50% coverage, and 596 lines would be necessary for over 90% coverage. The proof-of-concept HLA-iPSCs, which cover 6.1% of the Saudi population, successfully demonstrated pluripotency and the ability to differentiate into various cell types including beating cardiomyocytes and neuronal progenitors. The comprehensive genetic analysis corroborated that all identified variants in the derived iPSCs were inherently present in the original donor sample and were classified as benign according to the standards set by the ACMG.

**Conclusions:**

Our study sets a road map for introducing iPS-based cell therapy in the Kingdom of Saudi Arabia. It underscores the pragmatic approach of HLA-based iPSC banking which circumvents the limitations of autologous iPS-based cellular therapies. The successful generation and validation of iPSC lines based on the most prevalent HLA haplotype in the Saudi population signify a promising step toward broadening the accessibility and applicability of stem cell therapies and regenerative medicine in Saudi Arabia.

**Supplementary Information:**

The online version contains supplementary material available at 10.1186/s13287-023-03612-0.

## Introduction

Induced pluripotent stem cells (iPSCs) are a type of stem cell that can be generated from adult somatic cells by reprogramming them to a pluripotent state [1]. Human iPSCs can indefinitely proliferate in the lab and be directed to differentiate into derivatives of all three germ layers [1, 2]. These two characteristics make iPSCs an attractive source of cells for cell therapy [3, 4]. Upon their discovery, iPSCs were hailed as a promising alternative to human embryonic stem cells (hESCs), as they overcome the ethical problems associated with hESCs derivation and alleviate the risk of immunological rejection [1, 5]. However, it became evident that developing autologous iPS-based cell therapy products for every patient is a laborious process that is currently prohibitively expensive and time-consuming [6–8].

Alternatively, human leukocyte antigen (HLA)-based banking of iPSCs for allogeneic cell therapy became a more attractive option [9]. HLA-matched cell therapy has been widely employed for hematopoietic stem cell transplantation for patients with blood cancers and other hematological disorders [10, 11]. However, HLA loci are highly polymorphic; therefore, generating thousands of iPS lines would be impractical. To mitigate this, it has been previously proposed that the generation of iPS cell stocks from carefully selected donors who are homozygous for the most common HLA haplotypes found in a given population, could offer coverage for every patient in need and could allow for the development of off-the-shelf cell therapy products [12–16].

To evaluate the feasibility of HLA-based banking of iPSCs in Saudi Arabia, we analyzed the database of the Saudi Stem Cell Donor Registry (SSCDR), which is a registry established to facilitate patient-donor matching for hematopoietic stem cell transplantation. The SSCDR database contained 64,315 high-resolution HLA genotypes of registered Saudi citizens at the time of our analysis. We found that HLA-based banking of iPSCs may be a suitable strategy for pilot implementation and introduction of iPS-based cell therapy in Saudi Arabia. Additionally, we herein describe the establishment of the first two iPS lines from a Saudi donor who is homozygous for the HLA haplotype with the highest frequency in the population and provides maximal coverage. We describe the donor recruitment process, the reprogramming method to be used, and quality control tests that will be employed in the establishment of the HLA haplobank of iPSCs in Saudi Arabia.

## Materials and methods

### Haplotype frequency analysis

HLA haplotype frequencies in the Saudi Arabian population were estimated based on haplotype information stored in the Saudi Stem Cell Donor Registry (https://kaimrc.ksau-hs.edu.sa/?page_id=1481) database. This database, comprising 64,315 individuals at the time of analysis, was analyzed using the EM algorithm as implemented in Hapl-o-Mat v 1.1 (10.1007/978-1-4939-8546-3_19) to estimate population level haplotype frequencies using two digit resolution. The haplotype coverage was estimated as detailed in Álvarez-Palomo (10.1186/s13287-021-02301-0) using an iterative algorithm. In each iteration, the most frequent haplotype was identified and all matching individuals were counted and removed from the dataset before the next iteration on the remaining dataset. Importantly, the haplotype matching procedure was modified by considering each locus independently and allowing matches on either of the two possible alleles per locus. Matching was performed based on the loci A, B, and DRB1.

### Cellular reprogramming and iPS generation

#### Ethical approval

This study was approved by the Institutional Review Board of Ministry of National Guard - Health Affairs (Protocol# RJ20/134/J). Initial donor recruitment was done by the Saudi Stem Cell Donor Registry staff. Personal interview was conducted, and informed consents were obtained by the research team.

#### PBMCs isolation and enrichment of erythroid progenitors

Peripheral blood was collected from the donor into EDTA-containing blood collection tube and treated with RosetteSep™ Human Progenitor Cell Basic Pre-Enrichment antibody cocktail according to the manufacturer’s instructions (StemCell Technologies Catalog#15226). After PBMCs separation and isolation, 1 million cells were cultured for 8 days in StemSpan™ SFEM II medium (StemCell Technologies Catalog #09605) supplemented with 1X StemSpan™ Erythroid Expansion Supplement (StemCell Technologies Catalog #02692).

#### Transfection of erythroid progenitor cells

Expanded erythroid cells were reprogrammed with Episomal iPSC Reprogramming Kit (Thermofisher Catalog#A15960). Around 3 × 105 cells were electroporated with 1 μg of each episomal vector using Neon Transfection System (Thermofisher). The emerging ESC-like colonies were manually picked and transferred into 96-well plates coated with rhLaminin-521 (Thermofisher Catalog#A29248) in the cGMP-grade mTeSR™ Plus medium (StemCell Technologies Catalog #100-0276). iPSCs were dissociated using the enzyme-free cGMP-grade ReLeSR (StemCell Technologies Catalog # 100-0484) using 1:10–1:30 splitting ratio and incubated at 37 °C, 5% CO_2_, 20% O_2_ incubator.

### Molecular characterization of pluripotency and genomic integrity

#### Immunocytochemistry

Cells were fixed in 4% (w/v) paraformaldehyde for 15 min, permeabilized in PBS containing 0.1% (v/v) Triton X-100 for 10 min, and subsequently blocked in PBS containing 1% gelatin for 45 min. Cells were incubated with primary antibodies overnight at 4 °C and probed with the appropriate secondary antibodies for 1 h at room temperature (ThermoFisher Scientific). Primary and secondary antibodies were resuspended in 0.2% gelatin in PBS. The nuclei were counterstained with 1 μg /mL DAPI nuclear staining (Thermo Fisher Scientific).

#### Quantitative reverse transcription PCR (RT-qPCR)

Total RNA was extracted using RNeasy Kit (Qiagen Catalog# 74104) and reverse-transcribed using the High-Capacity cDNA Reverse Transcription Kit (Applied Biosystems™ Catalog# 4374966). The RT-qPCR assay was carried out using FastStart SYBR Green Master Mix (ROCHE) as described previously (Alsayegh et al., 2018).

#### In vitro differentiation

The generated iPSCs were differentiated into the three germ layers using the STEMdiff™ Trilineage Differentiation Kit (StemCell Technologies Catalog #05230).

#### Flow cytometry analyses

Cells were stained with OCT4, NANOG, SOX2, and cTnI antibodies diluted in 2% FBS in PBS for 30 min on ice protected from light with occasional vortexing. It was then washed with PBS and analyzed on BD FACS ARIA cell sorter. FITC-positive cells were measured in stained vs unstained cells.

#### Karyotyping

For G banding karyotyping, iPSC lines were treated with 0.3 μg/mL KaryoMAX™ Colcemid™ (1 μg) for 15 min, dissociated by TrypLE, and incubated in hypotonic solution (75 mM potassium chloride) at 37 °C for 20 min. iPSCs were then fixed in methanol/glacial acetic acid 3:1 and stored at 4 °C. At least 50 metaphases were karyotyped at the department of pathology and laboratory medicine (Ministry of the National Guard—Health Affairs).

#### Neural progenitor cells (NPCs) differentiation

The generation of central nervous system (CNS)-type neural progenitor cells (NPCs) from HLA-iPSCs was performed according to Monolayer Culture Protocol (STEMdiff™ SMADi Neural Induction Kit Catalog #08581).

#### Cardiomyocyte differentiation

The differentiation of hESCs toward beating cardiomyocyte was performed following STEMdiff™ Ventricular Cardiomyocyte Differentiation Kit (Stem Cell Technologies Catalog #05010) in accordance with the manufacturer instructions. In brief, iPSCs were detached using gentle cell dissociation reagent and seeded at 1.2 × 10^6^ cells/well on Matrigel-coated 12 well plates in presence of mTeSR™ Plus medium and 10 μM Y-27632. Subsequently, the differentiation was initiated by replacing culture medium with Cardiomyocyte Differentiation Medium A for 48 h. at 37 °C, 5% CO_2_. Cardiomyocyte Differentiation Medium B was added for another 48 h. Then, Cardiomyocyte Differentiation Medium C was replaced on day 4 and 6. We perform a full-medium change with Cardiomyocyte Maintenance Medium every other day up to 20 days.

#### Episomal plasmids screening

DNA was extracted using AllPrep DNA/RNA/ Mini Kit (Qiagen Catalog# 80204). PCR was performed using EBNA-1 primers that detect all five episomal plasmids (expected size 666 bp) according to manufacture guidelines (Thermo Fisher Scientific Catalog # A15960).

#### Mycoplasma detection

Mycoplasma contamination was assessed using LookOut^®^ Mycoplasma qPCR Detection (SIGMA) (Additional file 4: Fig. S2C).

#### Statistical analysis

RT-qPCR data are represented as mean ± standard deviation (SD). Statistical significance was determined in Student’s *t*-test (unpaired; two-tailed). A Bonferroni correction was applied to the *p*-value from multiple comparisons. *p < 0.05.

#### Short tandem repeats (STR) identity assay

Extracted genomic DNA from HLA-iPSCs and PBMCs was analyzed for 24 polymorphic STR markers using GenePrint 24 system (Promega, Madison, USA) following manufacturer’s protocol and was amplified using PCR and followed by ABI capillary electrophoresis. In this analysis, 24 autosomal STR were analyzed D8S1179, D21S11, D7S820, CSF1PO, D3S1358, THO1, D13S317, D16S539, D2S1338, D19S433, vWA, TPOX, D18S51, D5S818, D1S1656, D2S441, D10S1248, Penta E, Penta D, DYS391, D12S391, D22S1045 FGA and Amelogenin using ABI 3130/3500 Genetic Analyzer.

#### Whole-genome sequencing (WGS)

The Nextera library prep kit (Illumina) was used to prepare libraries for WGS resequencing on the Novaseq 6000 sequencer (Illumina). The short-read sequences obtained from a blood sample as control, as well as the two cell lines iPSC#1 and iPSC#2 were assessed with FastQC (Andrews, n.d.). Adapter and low-quality regions were trimmed with Trimmomatic v0.33 [17] using parameters: 2:30:10 LEADING:3 TRAILING:3 SLIDINGWINDOW:4:20 MINLEN:40, leaving 280, 262, and 245 million reads for the blood, iPSC#1 and iPSC#2 dataset, respectively. Trimmed reads were mapped to the human reference genome assembly UCSC hg38 analysis set using BWA mem [18], which yielded a median coverage between 23 and 26X. Duplicates were marked and read groups added with Picard tools. The donor sample as well as both generated cell lines were HLA genotyped using xHLA (https://www.pnas.org/doi/10.1073/pnas.1707945114). Single nucleotide polymorphisms were jointly called in all samples with GATK HaplotypeCaller [19] following GATK best practices recommendations as well as with GATK Mutect2. In case of Mutect, mapped reads from HLA-iPSC#1 and HLA-iPSC#2 were run separately as treatment while the blood sample was provided as normal reference. Single nucleotide polymorphism calls were then filtered requiring a minimal allele frequency of 20%. The obtained polymorphisms were then annotated using the Ensembl Variant Effect Predictor VEP [20]. Detected SNVs were tested for overlap with genes listed in the Catalogie of Somatic Mutations In Cancer (COSMIC) Cancer Gene Census (https://cancer.sanger.ac.uk/census) and the Shibata cancer gene panel (https://www.pmda.go.jp/files/000152599.pdf). Variants which were predicted to have a high impact on the aforementioned gene set were manually examined.

Structural variants were called with Delly v1.1.6 and Manta v1.6 in subtractive mode specifying the blood sample as control and both cell lines separately as treatments. Structural variants passing the quality filter for each caller and being classified as “precise” were retained. Variants call for each cell line from Delly and Manta were then compared using SURVIVOR [21] and only overlapping calls were retained for further analysis, allowing for at most 500 bp distance between break points. This procedure revealed no structural variants in HLA-iPSC#2 and one tandem duplication HLA-iPSC#1.

## Results

### Identification of HLA homozygous donors in the Saudi population

To identify potential homozygous donors, we examined the SSCDR HLA database for haplotype frequencies for HLA-A, HLA-B, and HLA-DRB1. Matching for these loci reduces allograft rejection and diminishes the use of immunosuppressive drugs [8]. Our analysis showed that generating iPS lines from homozygous donors for the ten most frequent haplotypes can be expected to offer haplotype matching for 12.94% of the Saudi population (Table 1). We also performed a 5-locus based analysis of the SSCDR database and compared our results with those described previously [31], which yielded a good correspondence (Additional file 3: Fig. S1) (Additional file 6: Table S1).

**Table 1:** Frequencies and cumulative frequencies of the 10 most common HLA-A, HLA-B, and HLA-DRB1 in the Saudi Arabian population

	Haplotype	Cumulative frequency (%)	Population frequency (%)
1	A*02:01g~B*50:01g~DRB1*07:01g	1.94	1.94
2	A*02:05g~B*50:01g~DRB1*07:01g	3.75	1.81
3	A*23:01g~B*50:01g~DRB1*07:01g	5.15	1.40
4	A*26:01g~B*08:01g~DRB1*03:01g	6.54	1.39
5	A*31:01g~B*51:01g~DRB1*13:01g	7.85	1.31
6	A*02:01g~B*07:02g~DRB1*15:01g	9.08	1.23
7	A*02:01g~B*51:01g~DRB1*04:02g	10.29	1.22
8	A*01:01g~B*41:01g~DRB1*07:01g	11.20	0.91
9	A*24:02g~B*08:01g~DRB1*03:01g	12.08	0.87
10	A*68:01g~B*08:01g~DRB1*03:01g	12.94	0.87

Linkage disequilibrium (LD) scores between alleles of some of the most frequent haplotype in the Saudi population were shown to be low in some cases, suggesting a considerable possibility of recombination [31, 22]. We therefore modified the matching procedure by splitting haplotypes into individual loci and performing matching per locus, where each of the two possible alleles can be counted as potential match. Iterative selection and removal of the haplotype matching the most individuals (see Materials and Methods for details) yields a very similar order of the top ten haplotypes (Table 2), including only one new haplotype A*02:01g~B*51:01g~DRB1*03:01g. The fraction of HLA matches offered by these ten haplotypes, however, increases significantly to 26.9% (Table 2).

**Table 2:** Top 10 haplotypes that maximize the coverage across the population, using allele-wise matching across both haplotypes

	Haplotype	Cumulative coverage (%)	Coverage (%)	Number of homozygous donors
1	A*02:01g~B*50:01g~DRB1*07:01g	6.10	6.08	72
2	A*02:05g~B*50:01g~DRB1*07:01g	9.40	3.72	64
3	A*26:01g~B*08:01g~DRB1*03:01g	12.30	3.05	39
4	A*31:01g~B*51:01g~DRB1*13:01g	14.90	2.77	57
5	A*02:01g~B*07:02g~DRB1*15:01g	17.30	2.81	27
6	A*02:01g~B*51:01g~DRB1*04:02g	19.60	2.71	44
7	A*23:01g~B*50:01g~DRB1*07:01g	21.80	3.24	56
8	A*02:01g~B*51:01g~DRB1*03:01g	23.70	2.51	8
9	A*24:02g~B*08:01g~DRB1*03:01g	25.40	2.24	21
10	A*01:01g~B*41:01g~DRB1*07:01g	26.90	1.90	35

**Fig. 1:**
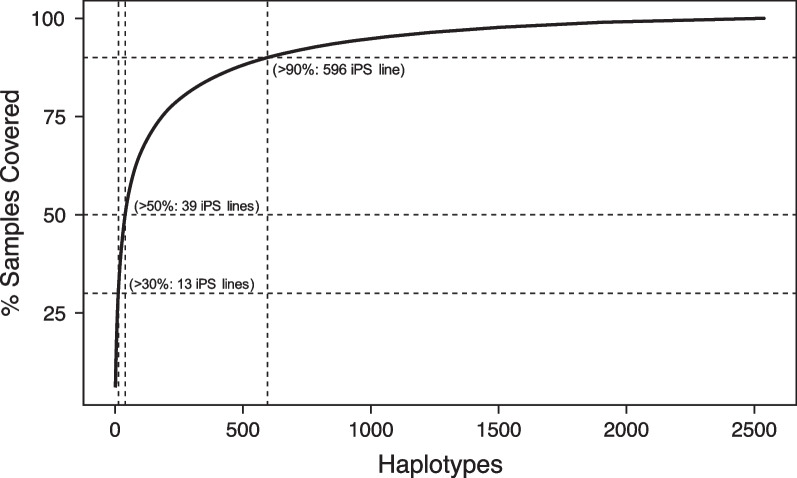
Estimated numbers of iPSC lines homozygous for HLA-A, HLA-B, and HLA-DRB1 (haplolines) and their coverage percentages for the Saudi population. The dotted lines mark 30, 50, and 90% coverage, for 13, 39 and 596 iPS lines, respectively

In extension, when using the maximized coverage approach, we found that a total of 13 haplotypes are estimated to have a match of 30% of the Saudi population (Fig. 1), versus 51 lines, when selecting by maximum population frequency and using haplotype-wise matching. The number of required haplotypes covering 50% of the population increases to 39 and 220 for the maximum-coverage allele-wise, and maximum-frequency haplotype-wise approach, respectively. Since the generation of 39 iPS lines to cover > 50% of the Saudi population is feasible, HLA-based banking of iPSCs may be a suitable strategy for the pilot implementation and introduction of iPS-based cell therapy in Saudi Arabia.

### Donor recruitment and derivation of HLA-haplobank iPS lines

In collaboration with the SSCDR, we identified a registered donor who is homozygous for the most common HLA haplotype (Table 1). This donor’s iPSCs would offer 6.1% coverage. The donor was initially contacted through the phone and upon approval, in-person interview was scheduled. After signing the informed consent, 10 ml peripheral blood sample was collected from the donor and erythroid progenitor cells (EPCs) were isolated expanded in culture for eight days. EPCs were chosen as the starting cell population for reprogramming due to their lack of DNA alterations and genomic structural variation including the absence of TCR/BCR genes recombination found in T-cells [23–25].

**Fig. 2:**
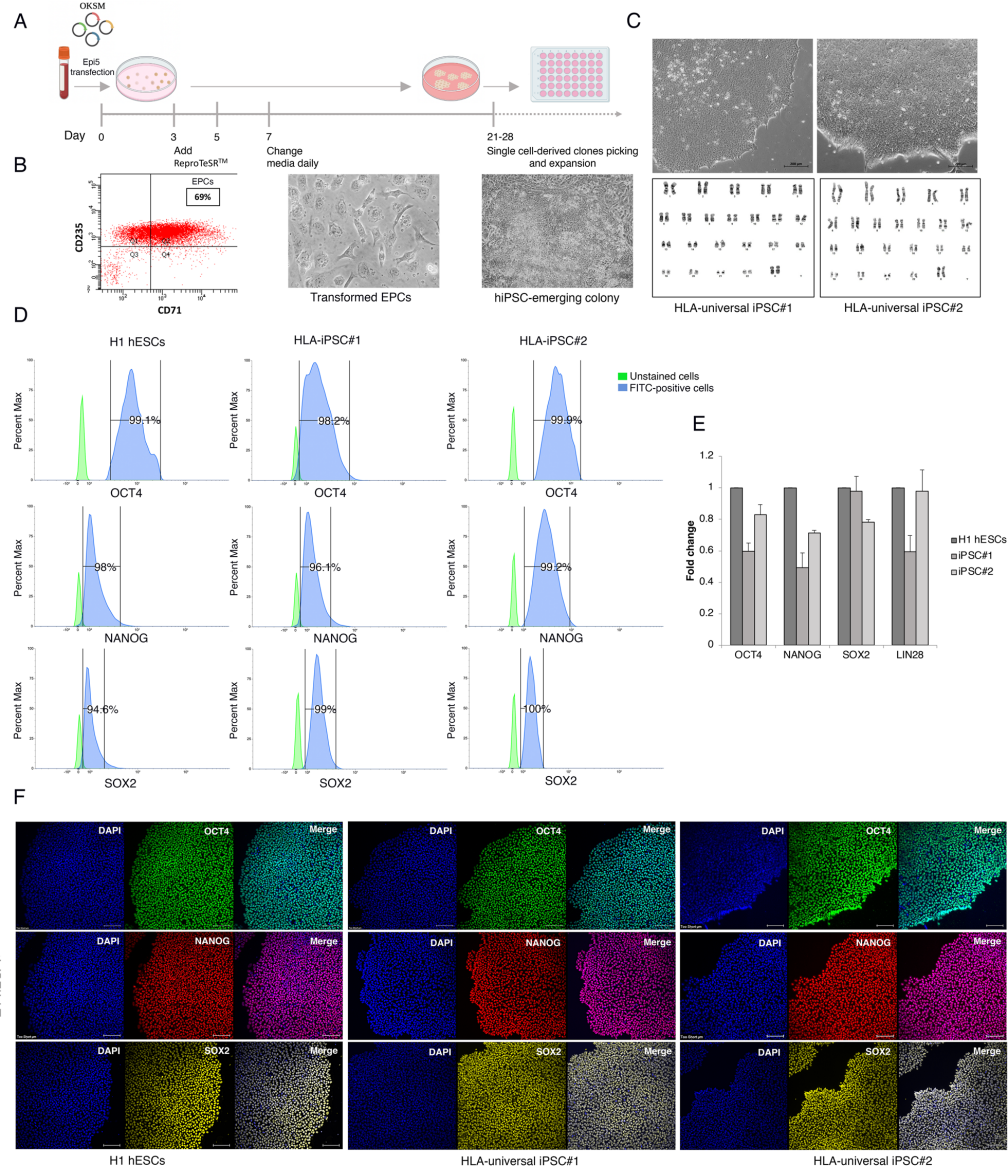
Generation and characterization of HLA-universal iPSCs. **A** Schematic representation of ReproTeSR™ and episomal reprogramming method. **B** Flow cytometry histogram for erythroid markers CD71 and CD235a EPCs culture on day 8 shows that ~ 70% of cells express the erythroid cell markers. Phase-contrast images of mesenchymal-to-epithelial transition and colonies appearance during reprogramming (days 11 to 28). **C** Top: representative images of HLA-universal iPS cell colonies generated from Erythroid Progenitor Cells exhibit more defined borders and compact morphology. Bottom: representative G-banded karyotype analysis for HLA-universal iPSCs shows normal karyotypes 46, XX. **D** Flow cytometry histograms of OCT4, NANOG, and SOX2 in HLA-universal iPSCs and H1 hESCs positive control. **E** Graph showing mRNA expression levels of pluripotency markers for the indicated iPSC lines presented as fold change relative to H1 hESC. Bars are median ± std of 3 biological replicates for each sample. **F** immunofluorescence staining of the pluripotency markers OCT4 (green), NANOG (red), and SOX2 (yellow), Nuclei were stained with DAPI (blue). Scale bar = 200 μm

Flow cytometry analysis showed that > 69% of cells stained positive for the erythroid markers CD71 and CD235a after EPCs expansion (Fig. 2A, 2B). On day 8 of expansion, reprogramming was initiated by electroporating EPCs with non-integrating episomal plasmids encoding OCT4, SOX2, KLF4, L-MYC, LIN28A, dominant-negative form of TP53, and EBNA1. At day 25 post-transfection, numerous embryonic stem cell (ESC)-like colonies were identified with typical ESC morphological characteristics (i.e., distinct borders, bright centers, tight-packed cells, and a high nucleus-to-cytoplasm ratio) (Fig. 2C). Such colonies were manually picked, expanded, and cryopreserved. Based on their ideal ESC-like morphology, two clones were chosen to be passaged and subjected to downstream pluripotency validation. The derived lines were registered in the Human Pluripotent Stem Cell Registry (hPSCreg) (https://hpscreg.eu/user/cellline/edit/KAIMRCi002-A, https://hpscreg.eu/user/cellline/edit/KAIMRCi002-B).

To assess the genomic integrity of HLA-iPSC#1 and #2, high-resolution G banding was performed after 12 passages in culture. More than 25 prometaphase spreads per clone were analyzed and showed normal female chromosomal number and structure (Fig. 2C). Short tandem repeats (STR) assay confirmed the matching identity of the isolated iPS lines and the donor EPCs (Additional file 4: Fig. S2A). Moreover, PCR analysis showed that the episomal plasmids were undetected in HLA-iPSC#1&2 after 12 passages (Additional file 4: Fig. S2B). Additionally, mycoplasma testing showed that the generated iPSC lines are mycoplasma-free (Additional file 4: Fig. S2C).

### Validation of iPSCs’ self-renewal and pluripotency

**Fig. 3:**
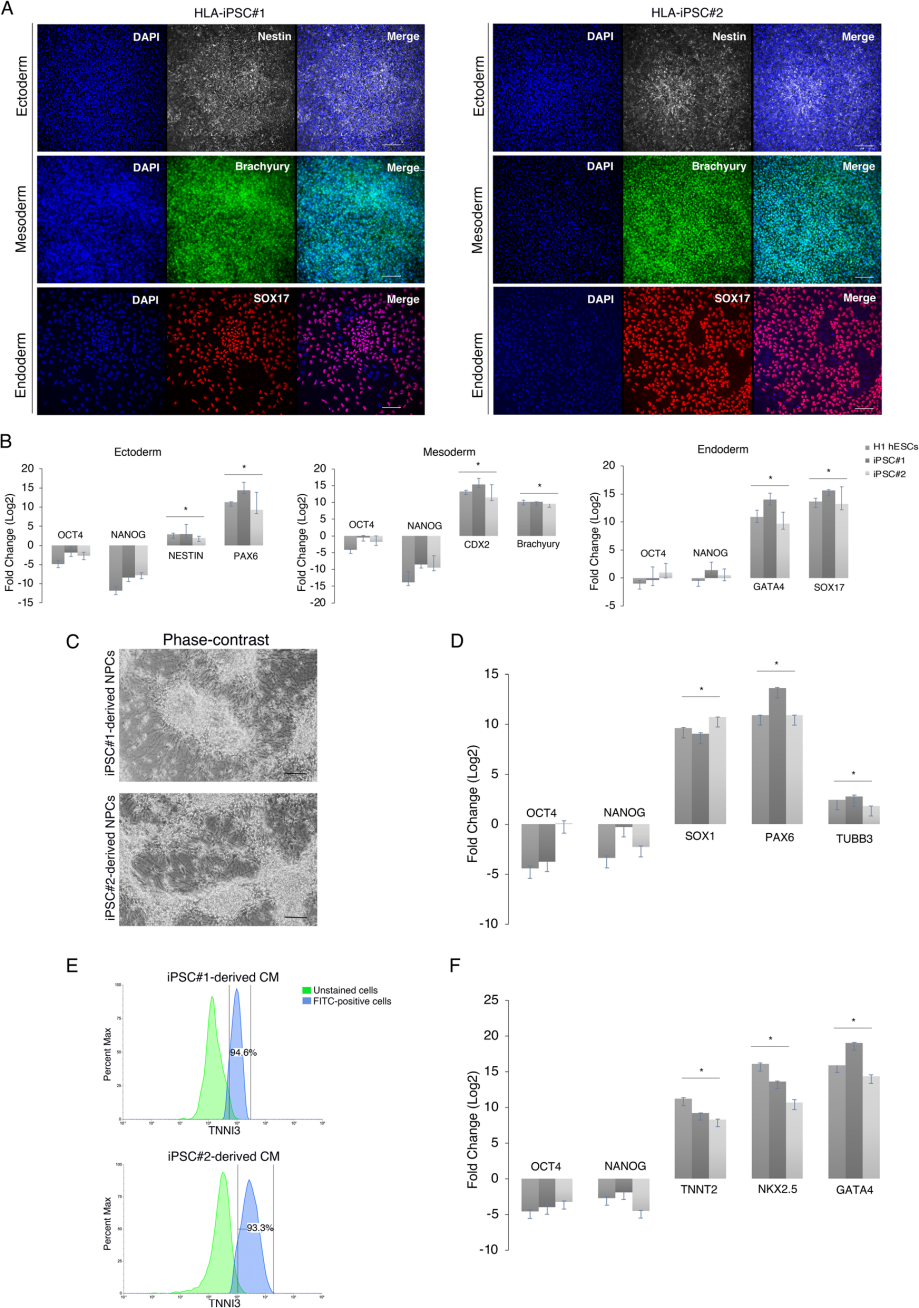
Differentiation potential of HLA-universal iPSCs. **A** immunofluorescence staining of specific markers for the three germ layers Ectoderm (NESTIN), Mesoderm (Brachyury), Endoderm (SOX17), Nuclei were stained with DAPI (blue). Scale bar = 200 μm. **B** Graphs showing mRNA expression levels of the lineage-specific markers for the three germ layers Mesoderm (*CDX2* and *Brachyury*), Endoderm (*GATA4* and *SOX17*), and Ectoderm (*NESTIN* and *PAX6*) presented as fold change relative to undifferentiated cells. Bars are median ± std of 3 biological replicates for each sample. Student’s t-tests, *p < 0.05. **C** Phase-contrast images of CNS-type NPC differentiation display typical NPC morphology 34 days post-differentiation. Scale bar = 200 μm. **D** Graph showing mRNA high expression levels of CNS-type NPCs markers SOX1, PAX6, and a low or negative expression of β-tubulin III presented as fold change relative to undifferentiated cells. Bars are median ± std of 2 biological replicates for each sample. Student’s t-tests, *p < 0.05. **E** Flow cytometry histograms display positive cTnI expression in iPSC-derived CM. (F) Graphs showing mRNA expression levels of cardiac markers *TNNT2*, *NKX2.4*, and *GATA4*. Bars are median ± std of 2 biological replicates for each sample. Student’s t-tests, *p < 0.05

Pluripotency markers OCT4, NANOG, and SOX2 were detected at the mRNA and protein levels in both clones. Flow cytometry histograms demonstrated that > 98% of cells stained positively for OCT4, > 96% for NANOG, and > 94% for SOX2 (Fig. 2D). Moreover, the derived iPSC lines displayed positive expression of *OCT4, NANOG, SOX2*, and *LIN28* by RT-qPCR (Fig. 2E) and OCT4, NANOG, and SOX2 by immunofluorescence (Fig. 2F). Direct in vitro differentiation to the three germ layers, mesoderm, definitive endoderm, and ectoderm was used to demonstrate the tri-lineage differentiation capacity. We observed a down-regulation of *OCT4* and *NANOG* and an upregulation of germ layer-specific markers by RT-qPCR (Fig. 3B). Immunostainings for the neural progenitor marker (NESTIN) indicated ectodermal differentiation. The positive expression of Brachyury, a member of the Tbox family, showed an early determination of mesoderm. We further assessed the presence of the endodermal marker SRY-Box Transcription Factor 17 (SOX17) (Fig. 3A). We, therefore, proved that the constructed HLA-universal iPSC lines possess bona fide characteristics of pluripotent stem cells. All performed quality control tests are summarized in Table 3.

**Table 3:** Summary of characterization tests performed on HLA-iPSC lines#1 and #2

Classification	Test	Results	Data
Morphology	Phase-contrast imaging	Typical primed pluripotent human stem cell morphology	Figure 2C, Top
Karyotype	G-banding	46, XX	Figure 2C, Bottom
Pluripotency status	Quantitative analysis (Flow cytometry, RT-qPCR)	Flow Cytometry: 98% OCT4, 96% NANOG, 99% SOX2 RT-qPCR: positive expression for OCT4, NANOG, SOX2, LIN28	Figure 2D, 2E
	Qualitative analysis (Immunocytochemistry)	Positive for the pluripotency markers: OCT4, NANOG, and SOX2	Figure 2F
Genetic identity	STR profiling	24 loci tested, all matched between donor EPCs and derived iPSC lines	Additional file 4: Fig. S2A
Verification of the absence of episomal vectors	PCR analysis	The five episomal DNA plasmids were undetected after 12 passages	Additional file 4: Fig. S2B
Mycoplasma testing	RT-qPCR	Negative	Additional file 4: Fig. S2C
Multilineage differentiation potential	Directed differentiation	RT-qPCR measurement of expression levels of ectoderm (*PAX6* and *NESTIN*), mesoderm (*BRACHYURY* and *CDX2*), and endoderm (*SOX17* and *GATA4*). Immunocytochemistry: Positive for NESTIN, BRACHYURY, and SOX17	Figure 3A, 3B
HLA typing	WGS	Homozygous at class I loci A, B, C, and class II loci DQB1 and DRB1, with only DPB1 being heterozygous	Figure 1
SV analysis	WGS; Manta + Delly + SURVIVOR	No SVs in iPSC#2; one tandem duplication in iPSC#1	Additional file 5: Fig. S3
SNP analysis	WGS; GATK, Mutect2	No high-impact SNP acquired during cell line establishment; SNPs in genetic background of the donor classified as benign	Additional file 8: Table S3 Table 4

Furthermore, the differentiation potential of the iPSC lines toward central nervous system (CNS)-type neural progenitor cells (NPCs) and beating cardiomyocyte was tested. CNS-type NPC differentiation induced a marked increase in key neuronal markers such as *SOX1*, *PAX6* and *TUBB3* (Fig. 3C, D).

The HLA-iPSC clones were also differentiated into cardiomyocytes (CMs) through a step-wise protocol. By day 15, the iPSC-derived CMs displayed spontaneous contractions, a unique functional property of pulsating heart muscles (Additional file 1: # movie). Flow cytometry histograms showed that > 94% of cells stained positively for Cardiac Troponin I (cTnI) (Fig. 3E). Moreover, RT-qPCR demonstrated that iPSC-derived CMs expressed the Cardiac Muscle Troponin T (*TNNT2*), myocardial markers NK2 Homeobox 5 (*NKX2.5*), and GATA family of zinc-finger transcription factor (*GATA4*) at mRNA levels (Fig. 3F).

### Whole-genome sequencing of generated iPS lines

To ascertain the genotype and whether the genomic integrity of the constructed iPSC lines was maintained during reprogramming and prolonged cultivation, we sequenced the genomes of the parental blood sample and the progenies iPSC#1 and iPSC#2 at passage 13. Genotyping of the HLA loci using the 23×–26× coverage read datasets confirmed homozygous status at class I loci A, B, C, and class II loci DQB1 and DRB1, with only DPB1 being heterozygous (A*02:01~A*02:01~B*50:01~B*50:01~C*06:02~C*06:02~DRB1*07:01~DRB1*07:01~DQB1*02:02~DQB1*02:02~DPB1*02:01~DPB1*04:01). Genomic variants were called in parental and progeny samples using GATK. This yielded a total of 5.4 million polymorphic sites with a mean genotype call rate of 99.2% and a heterozygosity ratio of 1.7. Out of the 4.3 million single nucleotide polymorphisms each sample had about 3.9 million polymorphic loci (Additional file 7: Table S2). We first focused on SNPs that were found polymorphic in all three samples to generate a high confidence variant set for the genetic background of the donor. We then examined any variants that might affect cancer-related genes based on the COSMIC Cancer Gene Census database and Shibata list as described previously (Yoshida et al. 2022) which involved 15 heterozygous SNVs and 4 homozygous SNVs in (see Materials and Methods for details). However, in the categories of sequence variants developed by the American College of Medical Genetics and Genomics (ACMG), we found that the 19 variants are almost certainly benign (Table 4). Thus, the direct link between these mutations and tumorigenicity was eliminated since the HLA universal donor was healthy at the time of iPSC generation.

**Table 4:** Examined variant loci in donor genetic background

Variant Locus	RefSeq	Gene	dbSNP	Genotype	ACMG Classification
1:150853154–150853154	ARNT (NM_001668.4):c.138-348C > T (p.?), Chr1(GRCh37):g.150825630G > A	ENSG00000143437	rs4072037	Heterozygote	Benign
1:155192276–155192276	MUC1 (NM_002456.6):c.66G > T (p.Thr22=), Chr1(GRCh37):g.155162067C > A	ENSG00000185499	rs4072037	Homozygote	Benign
12:6590204–6590204	CHD4 (NM_001273.5):c.3340 + 1261dup (p.?), Chr12(GRCh37):g.6699378dup	ENSG00000111642	rs58925722	Heterozygote	Benign
14:60658222–60658222	SIX1 (NM_005982.4):c.-9033T > C (p.?), Chr14(GRCh37):g.61124940A > G	ENSG00000126778	re10143202	Heterozygote	Benign
15:88253479–88253479	NTRK3-AS1 (NR_038229.1):n.749 + 1G > A, Chr15(GRCh37):g.88796710G > A	ENSG00000260305	rs1105693	Heterozygote	Benign
17:7667260–7667260	TP53 (NM_000546.6):c.*2348dup (p.?), Chr17(GRCh37):g.7570591dup	ENSG00000141510	rs34103303	Heterozygote	Benign
2:29221229–29221229	ALK (NM_004304.5):c.3516-394G > A (p.?), Chr2(GRCh37):g.29444095C > T	ENSG00000171094	rs1569156	Homozygote	Benign
3:10046723–10046724	FANCD2 (NM_001018115.3):c.1278 + 1del (p.?), Chr3(GRCh37):g.10088408del	ENSG00000144554	rs750338758	Heterozygote	Benign
3:149657502–149657502	WWTR1-AS1 (NR_040250.1):n.-708G > A, Chr3(GRCh37):g.149375289G > A	ENSG00000018408	rs6783790	Heterozygote	Benign
5:142771377–142771377	ARHGAP26 (NM_001135608.3):c.154 + 462T > C (p.?), Chr5(GRCh37):g.142150942T > C	ENSG00000145819	rs10042074	Heterozygote	Benign
5:143214087–143214087	ARHGAP26 (NM_001135608.3):c.2190C > T (p.Asn730 =), Chr5(GRCh37):g.142593652C > T	ENSG00000145819	rs258819	Homozygote	Benign
6:29943463–29943463	HLA-A (NM_002116.8):c.539T > A (p.Leu180X), Chr6(GRCh37):g.29911240T > A	ENSG00000206503	rs9260156	Heterozygote	Benign
6:29944251–29944252	HLA-A (NM_002116.8):c.751del (p.Asp251fs), Chr6(GRCh37):g.29912030del	ENSG00000206503	rs45576436	Heterozygote	Benign
6:41936060–41936060	CCND3 (NM_001760.5):c.759G > T (p.Glu253Asp), Chr6(GRCh37):g.41903798C > A	ENSG00000112576	rs33966734	Heterozygote	Benign
6:149716206–149716206	LATS1 (NM_004690.4):c.-141 + 1643G > A (p.?), Chr6(GRCh37):g.150037342C > T	ENSG00000131023	rs182844352	Heterozygote	Benign
7:116724769–116724770	MET (NM_000245.4):c.1201-6898del (p.?), Chr7(GRCh37):g.116364824del	ENSG00000105976	rs34822187	Heterozygote	Benign
7:152247986–152247986	KMT2C (NM_170606.3):c.2447dup (p.Tyr816X), Chr7(GRCh37):g.151945072dup	ENSG00000055609	rs150073007	Heterozygote	Benign
9:134029563–134029564	BRD3OS (NM_001355256.2):c.*2564del (p.?), Chr9(GRCh37):g.136894688del	ENSG00000235106	rs139424439	Heterozygote	Benign
X:41357831–41357831	DDX3X (NM_001356.5):c.*10112A > T (p.?), ChrX(GRCh37):g.41217084A > T	ENSG00000215301	rs6520743	Homozygote	Benign

Variant locus provided as [chromosome]:[bp], RefSeq transcript, Ensembl gene identifier, dbSNP variant identifier, genotype in donor (heterozygous/homozygous) and classification as per ACMG

In the second step, we tested whether the cell lines acquired new SNPs compared to the donor, using the donor sample as matched normal for the cell line samples. This approach yielded 1,610 and 1,888 SNPs for iPSC#1 and iPSC#2, respectively (Additional file 8: Table S3). None of the detected SNPs is predicted to have high impact with the majority classified as modifier.

While the subtractive analysis of structural variants (SVs) of donor vs. cell line did not detect newly acquired mutations in HLA-iPSC#2, it revealed a heterozygous tandem duplication on chromosome 16 (74,726,891 bp—74,727,373 bp) in HLA-iPSC#1 (Additional file 8: Fig. S3) which spans part of exon 3 of the Fatty Acid 2-Hydroxylase (FA2H) where it could lead to an alteration in the transcript. However, this gene is not part of the COSMIC Cancer Gene Census database or Shibata list rendering this variant benign.

## Discussion

Within only seven years of their initial derivation in 2007, iPSCs moved to clinical studies when a patient with age-related macular degeneration (AMD) was the first recipient of autologous iPS-derived retinal pigment epithelial cell sheet, in the world’s first in human clinical trial [4]. However, it became evident that the high cost and extended waiting time associated with autologous iPS-based cellular therapy, posed a significant hurdle to the advancement into the clinical domain [6–8].

One approach that was proposed to solve the time and cost problems is the creation of a hypo-immunogenic iPS cell line that evades the immune system. In this approach, iPS cells would be genetically modified to inactivate major histocompatibility complex (MHC) class I and II genes [26, 27]. However, to achieve this, multiple rounds of gene editing are required, which extends the time the cells are cultured, thus increasing the risk of acquiring mutations. Additionally, gene editing technologies like, CRISPR/Cas9 has been shown to introduce unintended genomic aberrations and may render the cells not useful for therapy [28, 29]. Even base and prime editing that does not involve double-strand breaks have recently been shown to induce significant genotoxicity in human cells [41].

Additionally, when HLA-I molecules are missing, NK cell responses may be increased in recipients [42]. Known as the “missing-self” hypothesis, the conventional consensus suggests that NK cells have the capability to identify and get rid of cells that don't display HLA class I molecules [43]. This concept has now evolved and is understood to be more intricate, encompassing various interactions between activating and inhibitory receptors on NK cells. The balance between these two types of signals dictates the behavior of the NK cells. Therefore, by influencing this balance in favor of inhibition, one can steer and control the response of NK cells. To evade detection and subsequent attacks by NK cells, the ectopic expression of immune-modifying molecules like HLA-E, HLA-G, and CD47 could be introduced, which adds to the complexity of achieving the sought after hypoimmunogenic line [44, 45].

Therefore, there has been an increased interest in HLA-based iPS banking in numerous countries [5, 14, 15, 30, 32, 46, 47]. In this study, we assessed the feasibility of creating an iPS haplobank in Saudi Arabia to develop clinical-grade iPS cell stocks, as the ultimate goal. In order to achieve this, we used the high-resolution HLA genomic database of the SSCDR, which at the time of analysis contained 64,315 registered donors, and assumed it was a representative sample of the Saudi population. We found that, the feasibility of HLA-based banking in Saudi Arabia is comparable to similar endeavors in other countries. We found that an iPS haplobank of the top 5 haplotypes that offer maximal coverage for the Saudi population would cover 17.30% of the population, which is close to the Spanish bank in which, the top 5 haplotypes cover 19.44%, but lower than the Korean estimation, in which the top 5 haplotypes cover 27.99% [14, 15]. This finding is in line with previous reports that showed a relatively high HLA genetic diversity among Saudis compared to other populations [31].

We found that an iPS haplobank generated from homozygous donors from the top 39 haplotypes would offer coverage of more than 50% of the Saudi population. This significant percentage may allow for many Saudi patients to benefit from iPS-derived cell therapies in the kingdom and therefore, it justifies the construction of the haplobank. In addition, streamlining the process of generating clinical-grade iPSCs will facilitate the establishment and future expansion of the bank to include additional haplotypes. Moreover, due to high level of consanguinity in the Saudi population, there is a considerable excess homozygosity, which may facilitate the identification of homozygous donors and haplobanking [40].

Due to the relatively high intra-population diversity in Saudi, we found that achieving higher coverage requires much larger cell stocks. Around 596 iPS line would be required to cover 90% of the population, and 2541 lines for 100% coverage. Even though we envisage that the establishing of an iPS cell stock to cover 30%-50% of the Saudi population is a feasible goal to introduce iPS-based cell therapy in the kingdom, achieving higher coverage percentage becomes increasingly cost-ineffective. Therefore, more research is needed to improve current methods of clinical-grade iPS generation to reduce cost and waiting time to make autologous cell therapy a possibility. Additionally, as we gain tighter control on the outcome of gene editing technologies, the creation of clinically relevant universal hypoimmunogenic iPS lines might become more feasible in the future.

To establish the workflow and initiate HLA-based banking in Saudi, we recruited the first donor and generated the first two clinically relevant iPS lines using defined feeder-free conditions. We chose EPCs to be the starting cell population for reprogramming. As opposed to human dermal fibroblasts, EPCs can be easily isolated and expanded from a simple ten ml blood sample and does not require painful skin biopsies. This is of particular importance in the donor recruitment process, as participants might be discouraged to donate if the procedure is invasive.

Additionally, EPCs are frequently replenished in the blood and therefore are less likely to accumulate environment-induced mutations like fibroblasts [33, 34]. Moreover, they lack the TCR/BCR genes recombination found in T-cells, making them a more attractive source of iPS cells. This is in addition to recent research demonstrating that erythroblasts-derived iPS cells are less likely to harbor genetic aberrations when compared to iPS cells from other sources [23, 25].

Eight days of expansion showed that around 69% of the cells were CD71^+^CD235a^+^ (Fig. 2B). The rest were CD71^−^CD235a^+^ and are more likely to be differentiated erythroblasts on their way to enucleation and are therefore, unamenable to reprogramming. Differentiated cells were particularly evident as red colored cells when EPCs were pelleted by centrifugation.

As an alternative to conventional retroviral-based cell reprogramming, non-viral, non-integrating plasmid-based reprogramming technique is more clinically relevant [35–37]. The reprogramming factors are delivered by vectors that contain oriP and EBNA-1, based on the Epstein-Barr Nuclear Antigen-1, which has demonstrated the ability to produce iPSCs highly efficiently without the potential risk of transgenic sequences being inserted into the target cell genome [38]. As opposed to other non-integrating reprogramming methods like Sendai virus and mRNA, episomal plasmids is the most cost-effective. Additionally, we found that these plasmids are readily removed from the reprogrammed cells as they were expanded, with most lines testing negative by end-point PCR by passage 12.

Following the expansion of EPCs, electroporation of the reprogramming episomal was carried out. ESC-like colonies appeared around 20–25 days post-transfection and were characterized by distinct borders, bright centers, tight-packed cells, and a high nucleus-to-cytoplasm ratio. The iPS clones were mechanically picked, expanded, and characterized for self-renewal and pluripotency in feeder-free culture conditions.

It is imperative to clarify that the current iPSC lines were not created in good manufacturing practice (GMP)-compliant laboratories; therefore, these lines will not be used for therapeutic purposes unless the required regulatory approvals were obtained and the lines are cleared. For developing clinical-grade HLA haplobank, KAIMRC is currently establishing its cell-processing-center in compliance with the updated GMP guidelines. Although the current HLA-iPSC#1 and iPSC#2 were generated inside research-grade labs, future haplobanking and clinical products will be derived and cryopreserved inside our GMP facility including re-derivation of the current HLA-iPSC lines to be clinical-grade. Re-derivation of human pluripotent stem cell lines inside GMP facilities has been done before. For instance, the H1 hESC line was re-derived and used as part of Astellas Pharma's phase II retinal pigment epithelium (RPE) trial, and re-derived H9 hESC line was used to generate dopaminergic neurons for a Parkinson's disease clinical trial by BlueRock Therapeutics [39].

## Conclusions

**Fig. 4:**
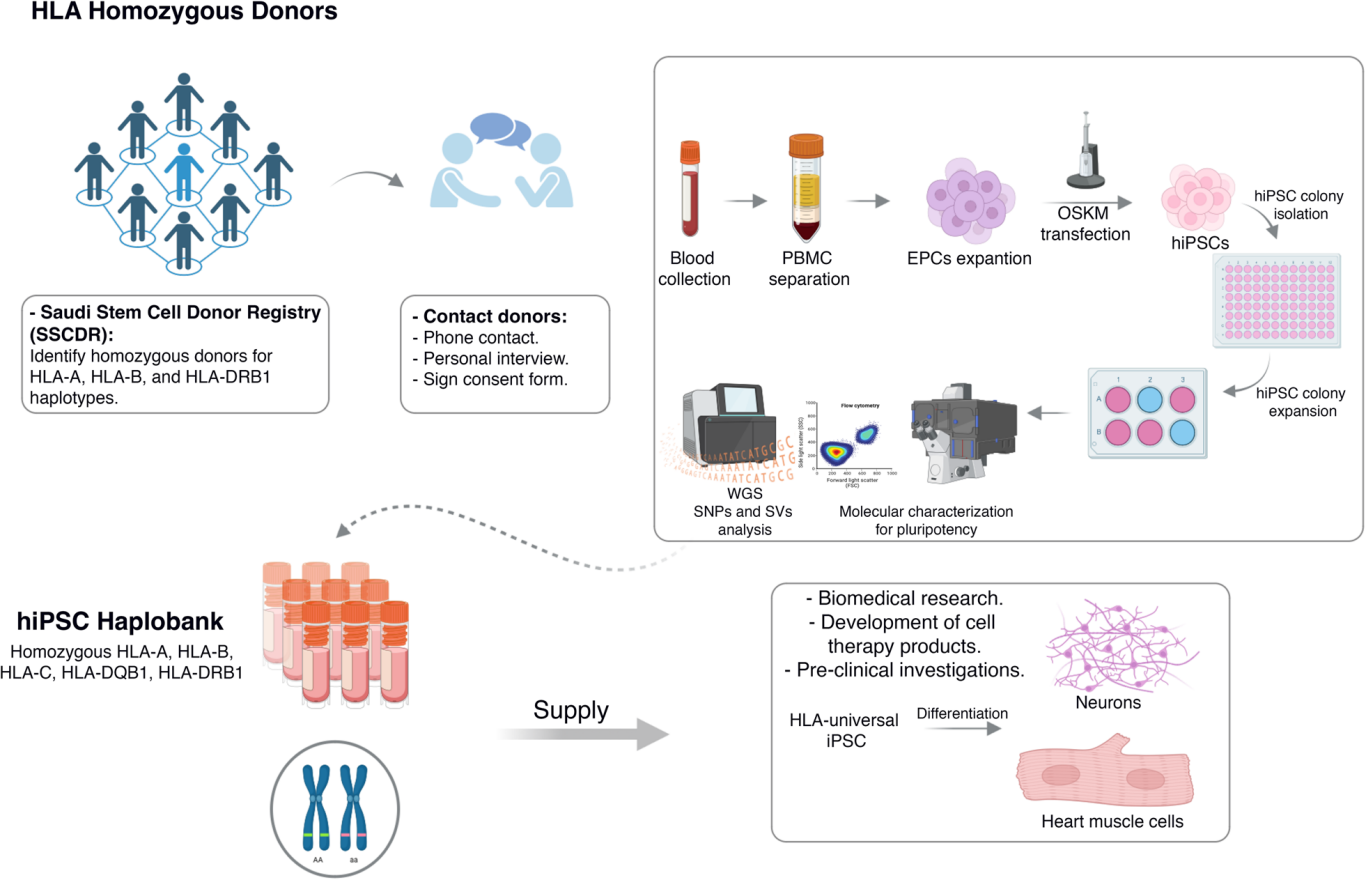
Graphical summary of the undergoing HLA-based banking in Saudi Arabia

Our study lays the foundation for the roadmap toward HLA-based banking of human induced pluripotent stem cells (iPSCs) in Saudi Arabia (Fig. 4). By interrogating the HLA database of the Saudi Stem Cell Donor Registry, we identified a subset of homozygous donors that could offer considerable coverage for the Saudi populace. Our analysis revealed that achieving 30% and 50% coverage necessitate the generation of 13 and 39 iPS lines, respectively. As a proof of principle, we successfully generated the first HLA-iPS line (2 clones), that offer 6.1% coverage of the Saudi population. By employing clinically relevant methodologies, the safety and quality of these iPSCs were maintained. Notably, whole-genome sequencing confirmed the genomic stability of the generated lines, hence alleviating concerns of high-risk mutations that may arise during reprogramming and expansion processes. Our study highlights the feasibility of HLA-based iPSC banking in Saudi Arabia and paves the way for a resilient infrastructure in regenerative medicine and personalized therapeutics.

### Supplementary Information

**Additional file 1. **HLA-iPS#2-derived cardiomyocytes exhibiting synchronous beating. The video was recorded at 10X magnification.

**Additional file 2. **Supplementary data.

**Additional file 3. Figure S1.** Comparison of cumulative 5-locus haplotype frequency of the SSCDR HLA database and the Alfraih et al.

**Additional file 4. Figure S2**. Cell lines authentication. (A) Short Tandem Repeat (STR) profiling guaranteed the genetic identity between the established iPSC lines and the donor EPCs. (B) PCR analysis detected the absence of the episomal plasmids in the indicated lines at passage 12. (C) RT-qPCR showing negative mycoplasma test in HLA-iPSC lines.

**Additional file 5. Figure S3**. Tandem duplication on chromosome 16 in cell line iPSC#1 visualized using IGV.

**Additional file 6. Table S1**. Comparison of 5-locus based haplotype frequencies based on the SSCDR database and KFSH&RC dataset (Alfraih et al. 2021).

**Additional file 7. Table S2**. Single nucleotide polymorphisms for donor blood sample and cell lines iPSC#1 and iPSC#2. The fraction of genotype calls matching with donor was calculated for biallelic SNPs.

**Additional file 8. Table S3**. Subtractive SNP calls for cell lines iPSC#1 and iPSC#2 vs. donor.

## Data Availability

All the presented data are available for consultation. Genomic dataset could be accessed through https://www.ncbi.nlm.nih.gov/bioproject/1047108 (Accession Number: PRJNA1047108).

## Abbreviations

SSCDR: Saudi Stem Cell Donor Registry
HLA: Human leukocyte antigen
EPCs: Erythroid progenitor cells
hESC: Human embryonic stem cell
iPSC: Induced pluripotent stem cells
hPSCreg: Human Pluripotent Stem Cell Registry
WGS: Whole-genome sequencing
SNPs: Single nucleotide polymorphisms
SVs: Structural variants
AMD: Age-related macular degeneration
GMP: Good manufacturing practice
RPE: Retinal pigmented epithelial cells

## References

[1] Takahashi K, Yamanaka S (2006). Induction of pluripotent stem cells from mouse embryonic and adult fibroblast cultures by defined factors. Cell.

[2] Yu J, Vodyanik MA, Smuga-Otto K, Antosiewicz-Bourget J, Frane JL, Tian S, Nie J, Jonsdottir GA, Ruotti V, Stewart R, Slukvin II (2007). Induced pluripotent stem cell lines derived from human somatic cells. Science.

[3] Singh VK, Kalsan M, Kumar N, Saini A, Chandra R (2015). Induced pluripotent stem cells: applications in regenerative medicine, disease modeling, and drug discovery. Front Cell Dev Biol.

[4] Mandai M, Watanabe A, Kurimoto Y, Hirami Y, Morinaga C, Daimon T, Fujihara M, Akimaru H, Sakai N, Shibata Y, Terada M (2017). Autologous induced stem-cell–derived retinal cells for macular degeneration. N Engl J Med.

[5] Taylor CJ, Peacock S, Chaudhry AN, Bradley JA, Bolton EM (2012). Generating an iPSC bank for HLA-matched tissue transplantation based on known donor and recipient HLA types. Cell Stem Cell.

[6] Habka D, Mann D, Landes R, Soto-Gutierrez A (2015). Future economics of liver transplantation: a 20-year cost modeling forecast and the prospect of bioengineering autologous liver grafts. PLoS ONE.

[7] Huang CY, Liu CL, Ting CY, Chiu YT, Cheng YC, Nicholson MW, Hsieh PC (2019). Human iPSC banking: barriers and opportunities. J Biomed Sci.

[8] Bravery CA (2015). Do human leukocyte antigen-typed cellular therapeutics based on induced pluripotent stem cells make commercial sense?. Stem Cells Dev.

[9] Taylor CJ, Bolton EM, Pocock S, Sharples LD, Pedersen RA, Bradley JA (2005). Banking on human embryonic stem cells: estimating the number of donor cell lines needed for HLA matching. Lancet.

[10] Gragert L, Eapen M, Williams E, Freeman J, Spellman S, Baitty R, Hartzman R, Rizzo JD, Horowitz M, Confer D, Maiers M (2014). HLA match likelihoods for hematopoietic stem-cell grafts in the US registry. N Engl J Med.

[11] Park M, Seo JJ. Role of HLA in hematopoietic stem cell transplantation. Bone Marrow Res. 2012;2012. 10.1155/2012/680841 PMC3467756 23082252

[12] Opelz G, Döhler B (2007). Effect of human leukocyte antigen compatibility on kidney graft survival: comparative analysis of two decades. Transplantation.

[13] Johnson RJ, Fuggle SV, O’Neill J, Start S, Bradley JA, Forsythe JL, Rudge CJ (2010). Kidney Advisory Group of NHS Blood and Transplant Factors influencing outcome after deceased heart beating donor kidney transplantation in the United Kingdom: an evidence base for a new national kidney allocation policy. Transplantation.

[14] Lee S, Huh JY, Turner DM, Lee S, Robinson J, Stein JE, Shim SH, Hong CP, Kang MS, Nakagawa M, Kaneko S (2018). Repurposing the cord blood bank for haplobanking of HLA-homozygous iPSCs and their usefulness to multiple populations. Stem Cells.

[15] Álvarez-Palomo B, García-Martinez I, Gayoso J, Raya A, Veiga A, Abad ML, Eiras A, Guzmán-Fulgencio M, Luis-Hidalgo M, Eguizabal C, Santos S (2021). Evaluation of the Spanish population coverage of a prospective HLA haplobank of induced pluripotent stem cells. Stem Cell Res Ther.

[16] Opelz G, Döhler B (2010). Pediatric kidney transplantation: analysis of donor age, HLA match, and posttransplant non-Hodgkin lymphoma: a collaborative transplant study report. Transplantation.

[17] Bolger AM, Lohse M, Usadel B (2014). Trimmomatic: a flexible trimmer for Illumina sequence data. Bioinformatics..

[18] Li H, Durbin R (2010). Fast and accurate long-read alignment with Burrows-Wheeler transform. Bioinformatics..

[19] McKenna A, Hanna M, Banks E (2010). The Genome Analysis Toolkit: a MapReduce framework for analyzing next-generation DNA sequencing data. Genome Res..

[20] McLaren W, Gil L, Hunt SE (2016). The ensembl variant effect predictor. Genome Biol..

[21] Jeffares DC, Jolly C, Hoti M (2017). Transient structural variations have strong effects on quantitative traits and reproductive isolation in fission yeast. Nat Commun..

[22] Jawdat D, Uyar FA, Alaskar A, Müller CR, Hajeer A (2020). HLA-A, -B, -C, -DRB1, -DQB1, and -DPB1 allele and haplotype frequencies of 28,927 Saudi stem cell donors typed by next-generation sequencing. Front Immunol.

[23] Araki R, Hoki Y, Suga T, Obara C, Sunayama M, Imadome K, Fujita M, Kamimura S, Nakamura M, Wakayama S, Nagy A (2020). Genetic aberrations in iPSCs are introduced by a transient G1/S cell cycle checkpoint deficiency. Nat Commun.

[24] Chou BK, Mali P, Huang X, Ye Z, Dowey SN, Resar L, Zou C, Zhang YA, Tong J, Cheng L (2011). Efficient human iPS cell derivation by a non-integrating plasmid from blood cells with unique epigenetic and gene expression signatures. Cell Res.

[25] Perriot S, Canales M, Mathias A, Du Pasquier R (2022). Generation of transgene-free human induced pluripotent stem cells from erythroblasts in feeder-free conditions. STAR Protoc.

[26] Xu H, Wang BO, Ono M, Kagita A, Fujii K, Sasakawa N, Ueda T, Gee P, Nishikawa M, Nomura M, Kitaoka F (2019). Targeted disruption of HLA genes via CRISPR-Cas9 generates iPSCs with enhanced immune compatibility. Cell Stem Cell.

[27] Kitano Y, Nishimura S, Kato TM, Ueda A, Takigawa K, Umekage M, Nomura M, Kawakami A, Ogawa H, Xu H, Hotta A (2022). Generation of hypoimmunogenic induced pluripotent stem cells by CRISPR-Cas9 system and detailed evaluation for clinical application. Mol Therapy-Methods Clin Dev.

[28] Fu Y, Foden JA, Khayter C, Maeder ML, Reyon D, Joung JK, Sander JD (2013). High-frequency off-target mutagenesis induced by CRISPR-Cas nucleases in human cells. Nat Biotechnol.

[29] Cradick TJ, Fine EJ, Antico CJ, Bao G (2013). CRISPR/Cas9 systems targeting β-globin and CCR5 genes have substantial off-target activity. Nucleic Acids Res.

[30] Yoshida S, Kato TM, Sato Y, Umekage M, Ichisaka T, Tsukahara M, Takasu N, Yamanaka S (2023). A clinical-grade HLA haplobank of human induced pluripotent stem cells matching approximately 40% of the Japanese population. Med.

[31] Alfraih F, Alawwami M, Aljurf M, Alhumaidan H, Alsaedi H, El Fakih R, Alotaibi B, Rasheed W, Bernas SN, Massalski C, Heidl A (2021). High-resolution HLA allele and haplotype frequencies of the Saudi Arabian population based on 45,457 individuals and corresponding stem cell donor matching probabilities. Hum Immunol.

[32] Gourraud PA, Gilson L, Girard M, Peschanski M (2012). The role of human leukocyte antigen matching in the development of multiethnic “haplobank” of induced pluripotent stem cell lines. Stem cells.

[33] Panther L, Ornelas L, Jones MR, Gross AR, Gomez E, Liu C, Berman B, Svendsen CN, Sareen D (2021). Generation of iPSC lines with high cytogenetic stability from peripheral blood mononuclear cells (PBMCs). BioRxiv.

[34] Kamimura S, Suga T, Hoki Y, Sunayama M, Imadome K, Fujita M, Nakamura M, Araki R, Abe M (2021). Insertion/deletion and microsatellite alteration profiles in induced pluripotent stem cells. Stem Cell Rep.

[35] Bang JS, Choi NY, Lee M, Ko K, Lee HJ, Park YS, Jeong D, Chung HM, Ko K (2018). Optimization of episomal reprogramming for generation of human induced pluripotent stem cells from fibroblasts. Anim Cells Syst.

[36] Yu J, Hu K, Smuga-Otto K, Tian S, Stewart R, Slukvin II, Thomson JA (2009). Human induced pluripotent stem cells free of vector and transgene sequences. Science.

[37] Okita K, Matsumura Y, Sato Y, Okada A, Morizane A, Okamoto S, Hong H, Nakagawa M, Tanabe K, Tezuka KI, Shibata T (2011). A more efficient method to generate integration-free human iPS cells. Nat Methods.

[38] Drozd AM, Walczak MP, Piaskowski S, Stoczynska-Fidelus E, Rieske P, Grzela DP (2015). Generation of human iPSCs from cells of fibroblastic and epithelial origin by means of the oriP/EBNA-1 episomal reprogramming system. Stem Cell Res Ther.

[39] Sullivan S, Fairchild PJ, Marsh SG, Müller CR, Turner ML, Song J, Turner D (2020). Haplobanking induced pluripotent stem cells for clinical use. Stem Cell Res.

[40] Chentoufi AA, Uyar FA, Chentoufi HA, Alzahrani K, Paz M, Bahnassy A, Elyamany G, Elghazaly A (2022). HLA diversity in Saudi population: high frequency of homozygous HLA alleles and haplotypes. Front Genet.

[41] Fiumara M (2023). Genotoxic effects of base and prime editing in human hematopoietic stem cells. Nat Biotechnol.

[42] Zhao W, Lei A, Tian L, Wang X, Correia C, Weiskittel T, Li H, Trounson A, Fu Q, Yao K, Zhang J (2020). Strategies for genetically engineering hypoimmunogenic universal pluripotent stem cells. iScience.

[43] Ljunggren HG, Kärre K (1990). In search of the “missing self”: MHC molecules and NK cell recognition. Immunol Today.

[44] Han X, Wang M, Duan S, Franco PJ, Kenty JH, Hedrick P, Xia Y, Allen A, Ferreira LMR, Strominger JL, Melton DA, Meissner TB, Cowan CA (2019). Generation of hypoimmunogenic human pluripotent stem cells. Proc Natl Acad Sci USA.

[45] Wang B, Iriguchi S, Waseda M, Ueda N, Ueda T, Xu H, Minagawa A, Ishikawa A, Yano H, Ishi T, Ito R, Goto M, Takahashi R, Uemura Y, Hotta A, Kaneko S (2021). Generation of hypoimmunogenic T cells from genetically engineered allogeneic human induced pluripotent stem cells. Nat Biomed Eng.

[46] Taylor CJ, Bolton EM, Bradley JA (2011). Immunological considerations for embryonic and induced pluripotent stem cell banking. Philos Trans R Soc B Biol Sci.

[47] Nakatsuji N, Nakajima F, Tokunaga K (2008). HLA-haplotype banking and iPS cells. Nat Biotechnol.

