# Supplementary figures and images for: HLA-based banking of induced pluripotent stem cells in Saudi Arabia
Source: Stem Cell Res Ther. 2023 Dec 18;14:374. doi: 10.1186/s13287-023-03612-0 (PMC10729375; doi:10.1186/s13287-023-03612-0)

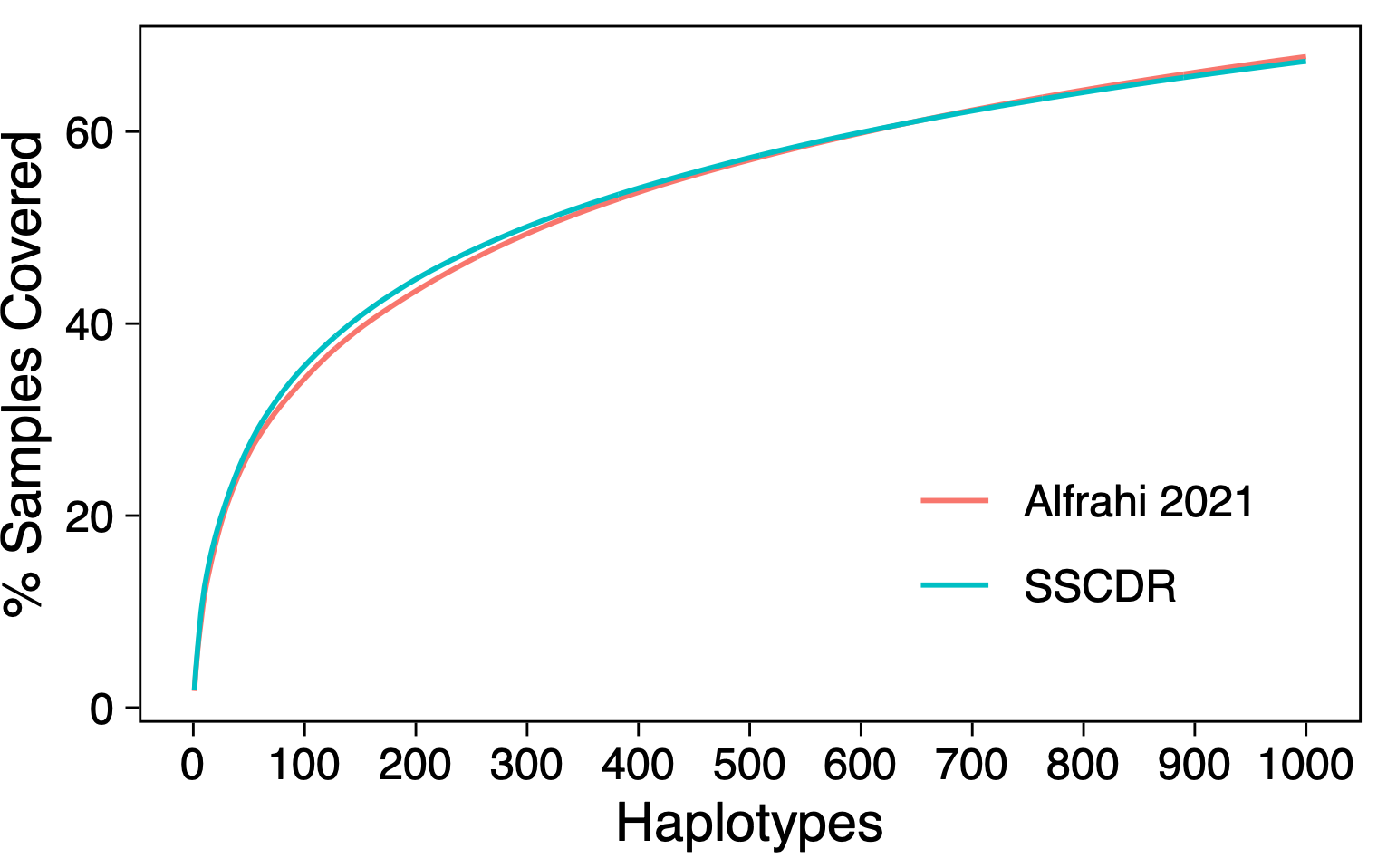

Supplement: Supplementary file 3 — Additional file 3. Figure S1. Comparison of cumulative 5-locus haplotype frequency of the SSCDR HLA database and the Alfraih et al. [file 13287_2023_3612_MOESM3_ESM.png]

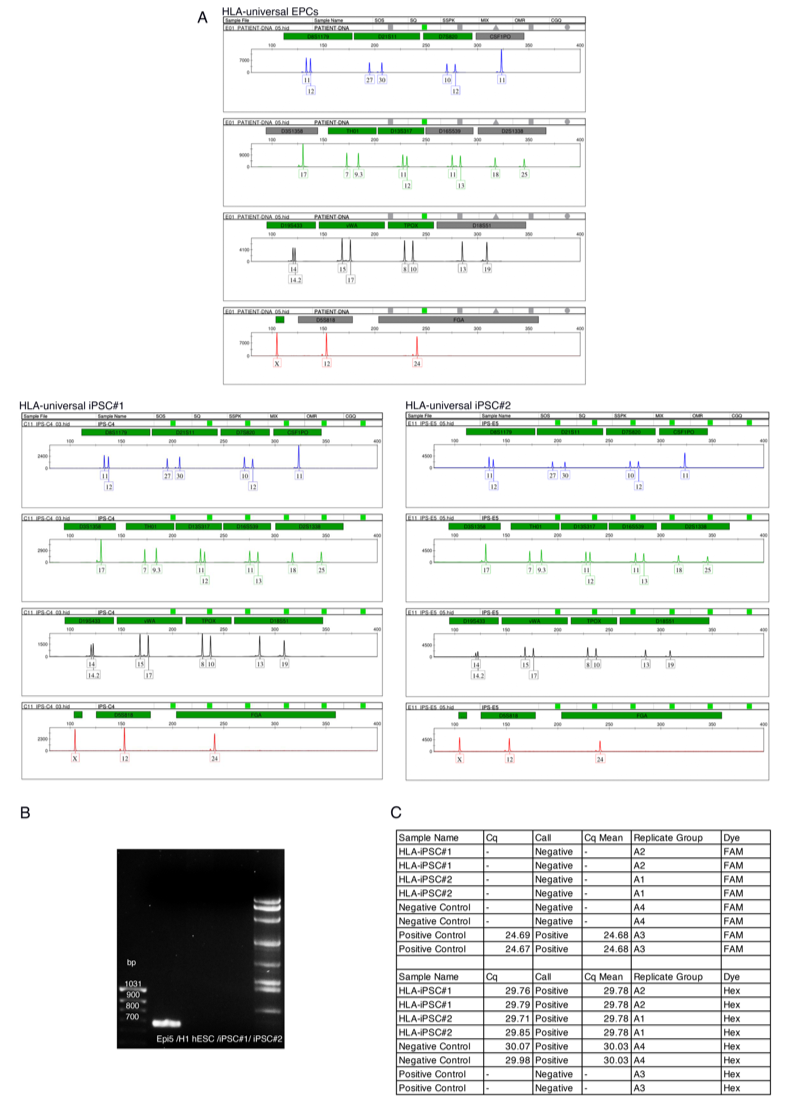

Supplement: Supplementary file 4 — Additional file 4. Figure S2. Cell lines authentication. (A) Short Tandem Repeat (STR) profiling guaranteed the genetic identity between the established iPSC lines and the donor EPCs. (B) PCR analysis detected the absence of the episomal plasmids in the indicated lines at passage 12. (C) RT-qPCR showing negative mycoplasma test in HLA-iPSC lines. [file 13287_2023_3612_MOESM4_ESM.png]

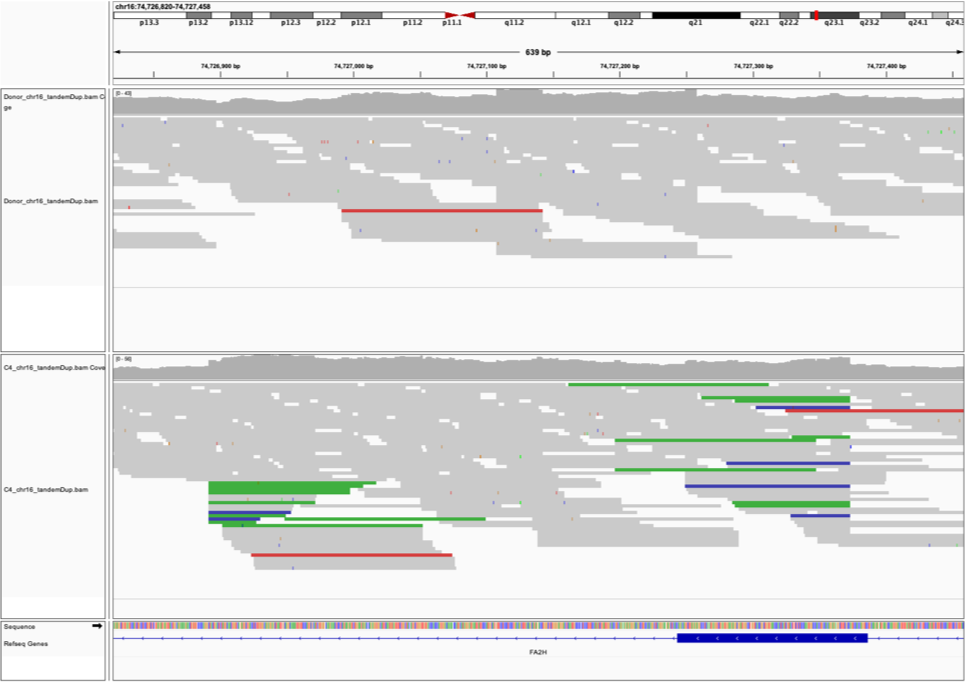

Supplement: Supplementary file 5 — Additional file 5. Figure S3. Tandem duplication on chromosome 16 in cell line iPSC#1 visualized using IGV. [file 13287_2023_3612_MOESM5_ESM.png]
